# Clinical characteristics and work-up of small to intermediate-sized pulmonary nodules in a Chinese dedicated cancer hospital

**DOI:** 10.20892/j.issn.2095-3941.2019.0028

**Published:** 2020-02-15

**Authors:** Xiaonan Cui, Daiwei Han, Marjolein A. Heuvelmans, Yihui Du, Yingru Zhao, Lei Zhang, Harry J.M. Groen, Geertruida H. de Bock, Monique D. Dorrius, Matthijs Oudkerk, Rozemarijn Vliegenthart, Zhaoxiang Ye

**Affiliations:** ^1^Department of Radiology, Tianjin Medical University Cancer Institute and Hospital, National Clinical Research Center for Cancer, Key Laboratory of Cancer Prevention and Therapy, Tianjin, Tianjin’s Clinical Research Center for Cancer, Tianjin 300060, China; ^2^Department of Radiology, University of Groningen, University Medical Center Groningen, Groningen 9713 GZ, The Netherlands; ^3^Department of Epidemiology, University of Groningen, University Medical Center Groningen, Groningen 9713 GZ, The Netherlands; ^4^Medisch Spectrum Twente, Department of Pulmonology, Enschede 7512 KZ, The Netherlands; ^5^Department of Thoracic Surgery, Tianjin Medical University Cancer Institute and Hospital, National Clinical Research Center for Cancer, Key Laboratory of Cancer Prevention and Therapy, Tianjin, Tianjin’s Clinical Research Center for Cancer, Tianjin 300060, China; ^6^Department of Pulmonary Diseases, University of Groningen, University Medical Center Groningen, Groningen 9713 GZ, The Netherlands; ^7^University of Groningen, University Medical Center Groningen, Groningen 9713 GZ, The Netherlands

**Keywords:** Lung nodule, diagnosis, computed tomography, pathology, China

## Abstract

**Objectives:** To evaluate the characteristics and work-up of small to intermediate-sized pulmonary nodules in a Chinese dedicated cancer hospital.

**Methods:** Patients with pulmonary nodules 4–25 mm in diameter detected *via* computed tomography (CT) in 2013 were consecutively included. The analysis was restricted to patients with a histological nodule diagnosis or a 2-year follow-up period without nodule growth confirming benign disease. Patient information was collected from hospital records.

**Results:** Among the 314 nodules examined in 299 patients, 212 (67.5%) nodules in 206 (68.9%) patients were malignant. Compared to benign nodules, malignant nodules were larger (18.0 mm *vs.* 12.5 mm, *P <* 0.001), more often partly solid (16.0% *vs.* 4.7%, *P <* 0.001) and more often spiculated (72.2% *vs.* 41.2%, *P <* 0.001), with higher density in contrast-enhanced CT (67.0 HU *vs*. 57.5 HU, *P* = 0.015). Final diagnosis was based on surgery in 232 out of 314 (73.9%) nodules, 166 of which were identified as malignant [30 (18.1%) stage III or IV] and 66 as benign. In 36 nodules (11.5%), diagnosis was confirmed by biopsy and the remainder verified based on stability of nodule size at follow-up imaging (*n* = 46, 14.6%). Among 65 nodules subjected to gene (EGFR) mutation analyses, 28 (43.1%) cases (EGFR19 *n* = 13; EGFR21 *n* = 15) were identified as EGFR mutant and 37 (56.9%) as EGFR wild-type. Prior to surgery, the majority of patients [*n* = 194 (83.6%)] received a contrast-enhanced CT scan for staging of both malignant [*n* = 140 (84.3%)] and benign [*n* = 54 (81.8%)] nodules. Usage of positron emission tomography (PET)-CT was relatively uncommon [*n* = 38 (16.4%)].

**Conclusions:** CT-derived nodule assessment assists in diagnosis of small to intermediate- sized malignant pulmonary nodules. Currently, contrast-enhanced CT is commonly used as the sole diagnostic confirmation technique for pre-surgical staging, often resulting in surgery for late-stage disease and unnecessary surgery in cases of benign nodules.

## Introduction

Lung cancer is the leading cause of tumor-associated mortality in China owing to diagnosis and treatment at relatively advanced stages^1^. Early detection, in combination with early resection of malignant pulmonary nodules, has the potential to improve lung cancer outcomes^2–4^.

With the widespread usage of low-dose multidetector computed tomography (CT), increasing numbers of small to intermediate-sized (mean diameter, 4 to 25 mm) pulmonary nodules have been detected, the majority of which are benign. Based on three rounds of screening in the National Lung Screening Trial (NLST)^5^, 96.4% of the findings (nodules > 4 mm) were false-positive. In a clinical setting, patients usually present with symptoms and larger-sized lung nodules (and masses)^6–8^. Due to differences in risk stratification, different management guidelines for nodules detected in lung cancer screening and clinical settings may be necessary.

In the past few years, considerable research attention has focused on optimizing management of pulmonary nodules detected in lung cancer screening sessions. The majority of these studies have investigated clinical diagnostic management within Western populations^8,9^. However, lung cancer types, genetic susceptibility and environmental factors are significantly different between Asian and Western populations. For instance, East Asian lung cancer patients, predominantly non-smokers with adenocarcinoma, have a much higher prevalence of epidermal growth factor receptor (EGFR) mutation than their Western counterparts (~30% and 7%, respectively)^10^. The potential influence of these differences on nodule characteristics and management is largely unknown.

In the current study, we have reviewed the clinical characteristics and work-up of small to intermediate-sized pulmonary nodules of patients admitted to a large dedicated cancer hospital in Northern China.

## Materials and methods

### Study population

The study was conducted at the Tianjin Medical University Cancer Institute and Hospital (TJMUCH), one of the most important specialized cancer centers in Northern China, which treats thousands of lung cancer patients each year. A consecutive search was performed using the PACS database on patients at TJMUCH receiving chest CT scans in 2013. The following inclusion criteria were applied: 1) all pulmonary nodules were newly detected in 2013 and 2) nodule diameters ranged from 4 to 25 mm. The final diagnosis of nodule nature (malignant or benign) was confirmed based on histological results or follow-up. Additionally, nodules were considered benign when they had disappeared, reduced in size by at least 30% or remained stable for at least 2 years on CT follow-up^11^. Exclusion criteria were as follows: 1) patients with suspected metastatic disease based on chest CT or histopathological findings and 2) nodules with unconfirmed results (for instance, no histology and inadequate follow-up period). Data on patients, nodules and clinical characteristics were collected from the hospital information system. Basic patient data included: 1) gender; 2) age; 3) smoking history; 4) cancer history; 5) family cancer history. Information on nodule characteristics included: 1) location (upper, middle or lower lobe); 2) size (average value of maximum long diameter and short diameter measured in the lung window); 3) shape (round, oval, irregular); 4) margin (smooth, lobulated, spiculated); 5) nodule type (non-solid, part-solid, solid); 6) density (attenuation value of the largest region of interest). Details of the assessment are described in **Supplementary Figure S1–S5**. Clinical characteristics included: 1) clinical diagnostic examination (CT, PET-CT, and biopsy); 2) surgery characteristics; 3) pathology results; (4) gene mutation information.

### CT protocol

Chest CT examinations were conducted using a Discovery CT750 HD (GE Medical Systems, Milwaukee, WI), Lightspeed 16 (GE Medical Systems) or Somatom Sensation 64 (Siemens Medical Solutions, Forchheim, Germany) CT system. The CT examination encompassed regions ranging from the pulmonary apex level to below the diaphragm. For scanning, tube voltage of 120 kVp with automatic tube current modulation was applied. For the GE CT system, reconstructed slice thickness and pitch were 1.25 mm and 0.984, respectively. For the Siemens CT system, reconstructed slice thickness and pitch were 1.5 mm and 0.95, respectively. The first CT examination was generally a non-contrast-enhanced CT. For further nodule evaluation, contrast-enhanced CT usually was performed at a later time. For contrast-enhanced CT, the iodine contrast agent Visipaque (Iodixanol, 270 mg/mL) was administered at a concentration of 1.5 mL/kg and injection rate of 2.5 mL/s. Contrast agents were administered intravenously through the upper extremity. Scanning was performed 70 s after the start of injection.

### Statistical analysis

Data are presented as numbers (%) for categorical variables. Pearson’s Chi-square test was used to examin differences in categorical variables between benign and malignant nodules. Normally distributed continuous data are presented as mean [standard deviation (SD)] and compared using Student’s *t*-test. Non-normally distributed continuous variables are presented as median [inter-quartile range (IQR)] and compared using a non-parametric test. *P*-values < 0.05 were indicative of statistical significance. All statistical analyses were performed using SPSS software version 20.0 (IBM, New York, US).

## Results

### Basic characteristics

**Figure 1:**
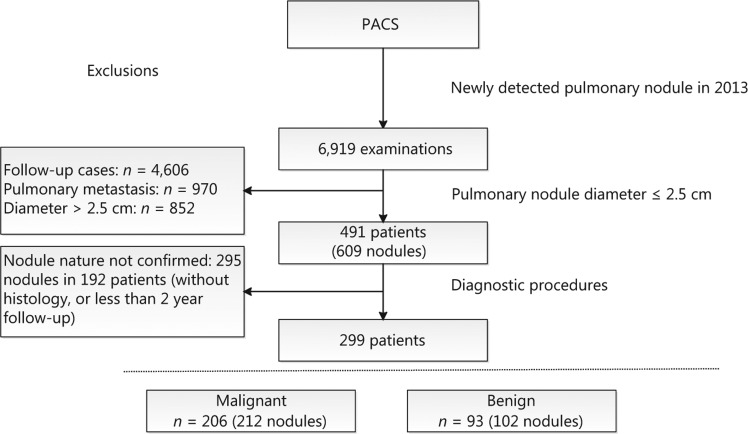
Flowchart of study population inclusion.

In total, 491 patients with 609 primary pulmonary nodules fulfilled the inclusion criteria. Owing to lack of diagnostic confirmation, 295 nodules in 192 patients were excluded, leading to the final inclusion of 299 patients with 314 nodules for analysis. Out of the 299 patients, 206 (68.9%) were diagnosed with lung cancer **(Figure 1)**. Within this patient group, 75 (72.1%) men and 16 (15.7%) women were smokers at the time of the study. Among 65 nodules subjected to EGFR mutation analysis, 13 (20%) were identified as EGFR19, 15 (23.1%) as EGFR21, and 37 (56.9%) as EGFR wild-type. Patient characteristics are presented in **Table 1**.

### Nodule evaluation

**Figure 2:**
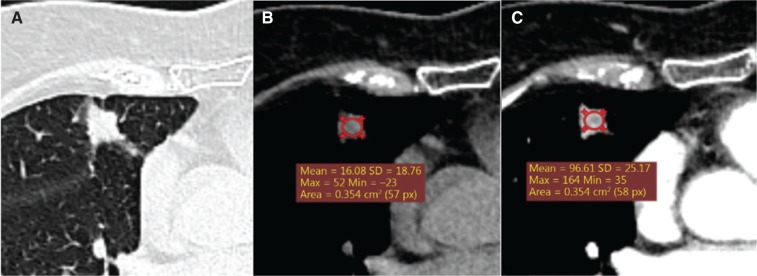
Patient example 1. Male, 64 years, a solid spiculated nodule (13 mm * 11 mm) located on the right upper lobe with the pleural indentation sign. The mean density of the nodule was 16 Hounsfield Units on CT without iodine contrast, and uneven enhancement 97 Hounsfield Units after contrast (A and B: non-contrast-enhanced CT. C: contrast-enhanced CT). Histology: Adenocarcinoma (stage I).

**Figure 3:**
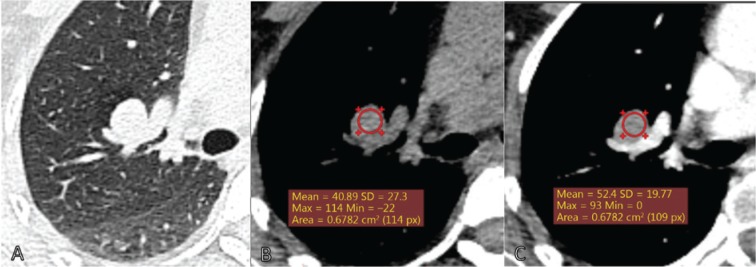
Patient example 2. Female, 58 years, a solid smooth nodule (18 mm * 14 mm) located on the right upper lobe and close to the vessel. The mean density of the nodule was 41 Hounsfield Units on CT without iodine contrast, and uniform enhancement 52 Hounsfield Units after contrast (A and B: non-contrast-enhanced CT. C: contrast-enhanced CT). Histology: Hamartoma.

In total, 212 of the 314 small to intermediate-sized nodules were malignant (67.5%). CT characteristics are presented in **Table 2**. The majority of nodules were solid (*n* = 262, 83.4%), among which 168 (64.1%) were malignant, while 34 out of the 36 (94.4%) partly solid nodules were malignant. The maximum diameter of malignant nodules was 18.0 mm (median, IQR: 14.0–22.0 mm), which was significantly larger than that of benign nodules (median 12.5 mm, IQR: 9.0–18.0 mm). Nodule margins differed significantly between malignant and benign nodules. Malignant nodules often were spiculated [*n* = 153 (72.2%)] while 46 (45.1%) of the benign nodules were smooth (*P <* 0.001). Nodule densities of benign and malignant nodules detected using non-contrast enhanced CT were similar. However, malignant nodules detected with contrast-enhanced CT had significantly higher density [67.0 HU (IQR: 51.0–81.0)] than benign nodules [57.5 HU (IQR: 34.5–77.0, *P =* 0.015)] **(Figure 2 and Figure 3)**.

### Management and histopathological diagnosis of pulmonary nodules

Histopathological diagnosis was obtained for 268 out of 314 (85.4%) nodules. In 232 (73.9%) cases, the nature of nodules was confirmed by surgery and 36 (11.5%) cases by biopsy. **Table 3** presents an overview of nodule work-up. Among the 102 benign nodules, 66 (64.7%) were confirmed by surgery, 6 (5.9%) by biopsy, and 30 (29.4%) by stable size or resolution at CT follow-up. Nodules confirmed as benign by surgery were significantly larger than those determined as benign after CT follow-up (14.0 mm *vs.* 9.0 mm, respectively, *P =* 0.001).

Before surgery, the majority of patients only received a contrast-enhanced CT scan after an initial non-contrast CT scan for nodule staging in both malignant [*n* = 140 (84.3%)] and benign [*n* = 54 (81.8%)] cases. Thirty-two of the patients with confirmed malignancy (19.3%) underwent a PET-CT scan before surgery, 2 (1.2%) underwent biopsy before surgery, and 12 (7.2%) underwent surgery immediately after the initial non-contrast CT scan. Among the resected lung cancers, 128 (77.1%) were diagnosed at stage I, 8 (4.8%) at stage II, 25(15.1%) at stage III, and 5 (3.0%) at stage IV (2 cases of pleural metastasis, 2 bone metastasis, 1 brain metastasis).

In total, 196 nodules were histologically confirmed as malignant. The majority of lung cancers were adenocarcinomas [*n* = 163 (83.2%)] and squamous cell carcinomas [*n* = 18 (9.2%)]. Among the 72 resected benign nodules, the most common histological diagnosis was hamartoma [*n* = 20 (27.8%)]. Histopathological results are shown in **Table 4**.

## Discussion

This study systematically describes the clinical characteristics and work-up of small to intermediate-sized primary pulmonary nodules in a Chinese clinical population based on data from one of the leading cancer hospitals in North China. We evaluated pulmonary nodules based on patient, nodule, and clinical characteristics.

### Patient characteristics

In our cohort, 67.5% of the clinically detected nodules were malignant. This lung cancer rate is obviously higher than that reported from community-based lung cancer screening using low-dose CT in China (malignancy rate between 0.6% and 1.5%)^3,12,13^. Moreover, relative to studies on other clinical cohorts, the percentage of malignant nodules in our analysis was considerably high^9^. This could be attributable to our hospital being a dedicated cancer center to which most patients are referred because of suspicious nodules. In fact, 192 patients with 295 nodules were excluded owing to unconfirmed diagnosis **(Figure 1)**. We lost contact with patients once they left our hospital, which presents a major problem in the long-term management of patients with pulmonary nodules in China.

Mean age at lung cancer diagnosis was similar to that reported in an earlier Chinese multi-institutional registry (CMIR) study (59.5±10.2 *vs.* 58.9±10.7)^3^. Smoking is the main cause of lung cancer, accounting for 80% of the male lung cancer burden and at least 50% of the female lung cancer burden worldwide^14^. However, in China, more than 30% of patients with lung cancer are non-smokers^15–17^. In our cohort, the proportion of smokers among female patients (15.7%) was substantially lower than that among male patients (72.1%). Several studies suggest second-hand smoking and gene mutations are risk factors for lung cancer in female non-smokers^18–20^. The EGFR mutation rate in our study was > 43%, which was significantly higher than that recorded in the Western population (approximately 7%)^10^.

### CT evaluation of pulmonary nodules

Radiologists generally estimate lung cancer probability on the basis of nodule CT characteristics. In our study, partly solid nodules showed a high probability of malignancy (94.4%), consistent with data from earlier screening studies^21–23^. For solid nodules, pre-surgical diagnosis was usually based on contrast-enhanced CT. Malignant nodules generally showed a large density difference between contrast-enhanced and non-enhanced CT, which was negligible or smaller for benign nodules. In our patients, contrast enhancement on CT was one of the most important differentiators of malignant and benign nodules before surgery.

Nodule margin is another important feature in determining nodule malignancy risk. Malignant nodules tend to be spiculated and lobulated while benign nodules are more likely to be smooth. In our cohort, 5.0% of malignant nodules were smooth, 22.6% were lobulated and 72.2% spiculated (*P <* 0.001, compared to margins of benign nodules). Similar findings have been reported previously^24–27^.

### Nodule diagnosis and management

One of the most important objectives of nodule work-up is to diagnose malignant nodules as rapidly and accurately as possible while minimizing the number of surgically removed benign nodules and late-stage lung cancers. Among the 232 resected nodules, 166 (71.6%) were malignant and 66 (28.4%) were benign. Only 19.3% lung cancer patients in our study underwent PET-CT prior to surgery, which is significantly lower than previous reports (55.0%)^9^. Instead, multiple body part CT and MRI scans were often performed for staging. Thus contrast-enhanced CT was the main imaging examination technique employed prior to surgery. This is consistent with work-up guidelines in China based on expert opinions, the Diagnosis and Treatment for Lung Nodules (2016 Version)^21^, which provides a choice of contrast-enhanced CT, PET-CT, and biopsy. Among the benign nodules, only 30 (29.4%) were examined using CT follow-up in our study. The percentage of resected benign nodules was high, since 59 (57.8%) of all benign nodules were considered high risk according to the China National Guideline of Classification^21^. Furthermore, the diameters of resected benign nodules were generally larger than 10 mm. Considering the relatively high resection rates of benign nodules and late-stage lung cancers (stage III or IV) in our study, it appears essential to refine the diagnostic process by using PET-CT to avoid unnecessary surgery. At the same time, excessive application of PET-CT will increase the financial burden for patients. To reach a compromise, more clinical research and expert consensus are required for optimization of future guidelines for China.

Our study population may reflect a high degree of selection bias, since our patients were sourced from a dedicated cancer hospital, with most being referred due to suspicion of malignant nodules. In addition, we only included patients with available pathology results or more than 2 years of follow-up. Several patients with small nodules that were considered benign did not receive imaging follow-up in our center and were therefore excluded. These factors may explain the relatively high rate of malignancy in our study population relative to other studies. Our data did not include patient information on ‘second-hand smoking’ and therefore, the effect of passive smoking was not estimated. Moreover, the current report is based on retrospective analysis of patient data from a single center and a multicenter prospective study is lacking.

## Conclusions

In conclusion, this study systematically describes the characteristics and work-up of clinically detected small to intermediate-sized pulmonary nodules in a Chinese population. CT-derived nodule features effectively assisted in diagnosis of malignant pulmonary nodules. Pre-surgery lung cancer risk estimation and staging are largely dependent on contrast-enhanced CT. PET-CT and biopsy were infrequently used for pre-surgical examination, which may explain the relatively high rate of resected late-stage lung cancers and benign nodules. Future research should focus on improvement of long-term management of patients with pulmonary nodules and follow-up strategies along with optimization of preoperative work-up.

## Figures and Tables

**Table 1:** Patient characteristics

Characteristics	Total	Malignant	Benign	*P*
Gender, *n* (%)				0.803
Male	153 (51.2)	104 (50.5)	49 (52.7)	
Female	146 (48.8)	102 (49.5)	44 (47.3)	
Age in years, mean (SD)	57.7 (10.7)	59.5 (10.2)	53.6 (10.8)	<0.001
Smoking history*, *n* (%)				0.415
Non smoker	157 (52.5)	112 (54.4)	45 (48.4)	
Smoker	120 (40.1)	91 (44.2)	29 (31.2)	
Pack-year smoking history*, median (IQR)	45.0 (20.0–80.0)	45.0 (20.0–80.0)	22.5 (15.0–45.0)	0.039
Family cancer history*, *n* (%)				0.299
No	225 (75.3)	168 (81.6)	57 (61.3)	
Yes	52 (17.4)	35 (17.0)	17 (18.3)	
Clinical symptoms*, *n* (%)				0.342
No	155 (51.8)	110 (53.4)	45 (48.4)	
Yes	122 (40.8)	93 (45.1)	29 (31.2)	
COPD, *n* (%)				0.514
No	247 (82.6)	168 (81.6)	79 (84.9)	
Yes	52 (17.4)	38 (18.4)	14 (15.1)	
EGFR mutation				
EGFR 19	13 (20.0)	13 (20.0)	-	
EGFR 21	15 (23.1)	15 (23.1)	-	
EGFR wild type	37 (56.9)	37 (56.9)	-	

*A total of 22 outpatients with no information on smoking, cancer history, family cancer history, and clinical symptoms. SD, standard deviation; IQR, interquartile range.

**Table 2:** CT characteristics of pulmonary nodules

Characteristics	Total	Malignant	Benign	*P*
Nodule type, *n* (%)				0.001
Non-solid	16 (5.1)	10 (4.7)	6 (5.9)	
Part-solid	36 (11.5)	34 (16.0)	2 (4.7)	
Solid	262 (83.4)	168 (79.2)	94 (92.2)	
Nodule size (mm)				<0.001
Median (IQR)	16.0 (12.0–20.0)	18.0 (14.0–22.0)	12.5 (9.0–18.0)	
Location, *n* (%)				0.886
Upper lobe	178 (56.7)	122 (57.5)	56 (54.9)	
Middle lobe	28 (8.9)	19 (9.0)	9 (8.8)	
Lower lobe	108 (34.4)	71 (33.5)	37 (36.3)	
Margin, *n* (%)				<0.001
Smooth	57 (18.2)	11 (5.2)	46 (45.1)	
Lobulated	62 (19.7)	48 (22.6)	14 (13.7)	
Spiculated	195 (62.1)	153 (72.2)	42 (41.2)	
Nodule density (HU), median (IQR)				
CT density unenhanced	26.0 (15.0–34.0)	27.0 (20.0–34.5)	26.5 (15.0–35.0)	0.481
CT density enhanced*	64.0 (47.0–80.0)	67.0 (51.0–81.0)	57.5 (34.5–77.0)	0.015
Difference value*	38.0 (21.9–54.0)	42.0 (26.5–54.5)	28.0 (8.5–46.3)	0.001

*n = 76 without contrast-enhanced CT examination.

**Table 3:** Diagnostic work-up of pulmonary nodules

Variable	Total	Malignant	Benign
Nodule nature confirmed by:			
Surgery	232 (73.9)	166 (78.3)	66 (64.7)
Biopsy	36 (11.5)	30 (14.2)	6 (5.9)
CT follow-up	46 (14.6)	16 (7.5)	30 (29.4)
Procedure prior to surgery, *n* (%)			
Contrast-enhanced CT	194 (83.6)	140 (84.3)	54 (81.8)
CT FU	18 (7.8)	16 (9.6)	2 (3.0)
PET CT	38 (16.4)	32 (19.3)	6 (9.1)
Biopsy	2 (0.8)	2 (1.2)	0 (0.0)
Only non-contrast enhanced CT	19 (8.2)	12 (7.2)	7 (10.6)
Stage of malignancy confirmed by surgery*, *n* (%)			
I	-	128 (77.1)	
II	-	8 (4.8)	
III	-	25 (15.1)	
IV	-	5 (3.0)	

*According to TNM 7^th^ version.

**Table 4:** Histopathology results

Characteristics	Pathology	No. of nodules (%)
Benign (*n* = 72)*	Inflammatory pseudotumor	13 (18.1)
	Granuloma	8 (11.1)
	Hamartoma	20 (27.8)
	Tuberculosis	13 (18.1)
	Sclerosing hemangiomas	6 (8.3)
	Sarcoidosis	1 (1.4)
	Pulmonary nodular lymphoid hyperplasia	3 (4.2)
Malignant (*n* = 196)*	Adenocarcinoma	163 (83.2)
	Squamous cell	18 (9.2)
	Small cell	5 (2.6)
	Large cell	1 (0.5)
	Carcinoid	3 (1.5)
	Clear cell	1 (0.5)

*Detailed information on histopathologic subtype was missing for 5 malignant and 8 benign nodules.
