# Supplementary material for: Clinical characteristics and work-up of small to intermediate-sized pulmonary nodules in a Chinese dedicated cancer hospital
Source: Cancer Biol Med. 2020 Feb 15;17(1):199–207. doi: 10.20892/j.issn.2095-3941.2019.0028 (PMC7142837; doi:10.20892/j.issn.2095-3941.2019.0028)
Supplement: Supplementary file 1 [file cbm-17-199-s001.pdf]

Supplementary materials

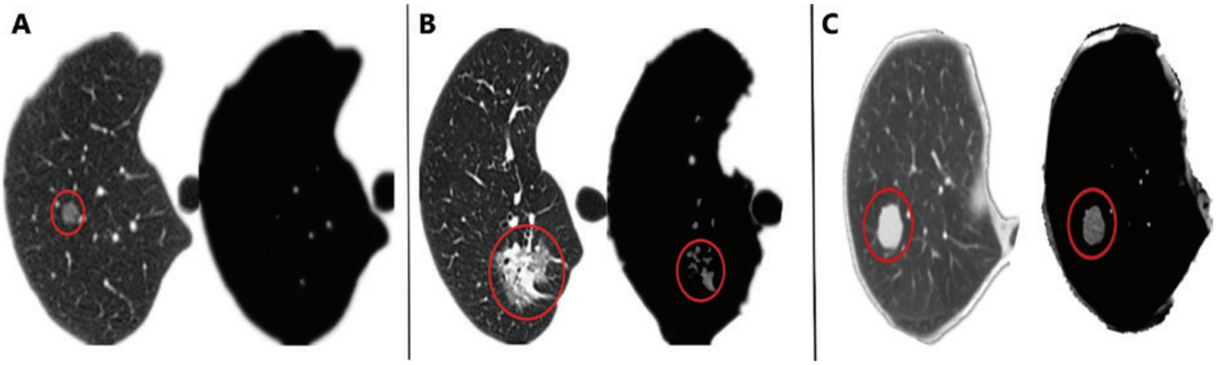

Figure S1 Type (non-solid nodule and part-solid nodule). (A) Non-solid nodule (NSN): nodule as a focal area of increased lung attenuation viewed on CT lung window settings (width, 1,450 HU; level, −500 HU), without a solid component viewed on mediastinal window setting (width, 350 HU; level, 40 HU). (B) Part-solid nodule (PSN): nodule with lesions viewed on CT lung window settings, and with a relatively smaller solid component viewed on mediastinal window setting. (C) Solid nodule (SN): nodule viewed both on CT lung window settings and mediastinal window setting with similar size.

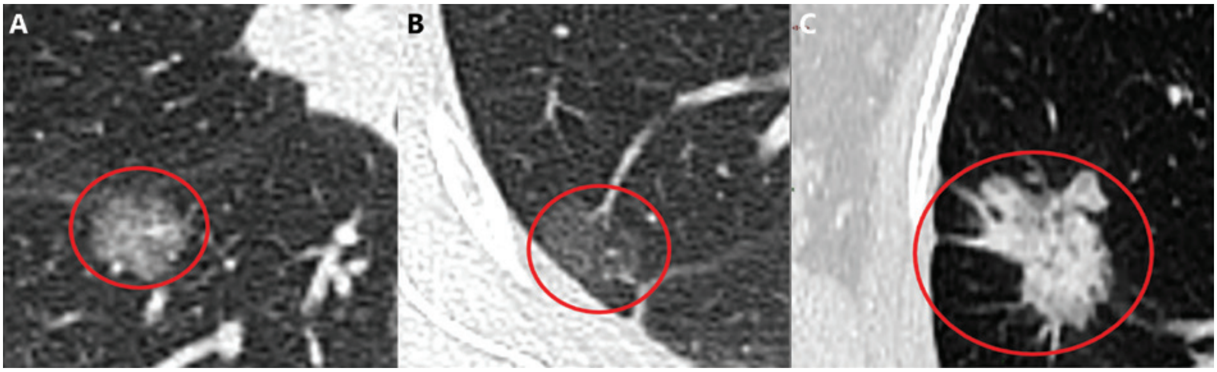

Figure S2 Shapes (round, oval, irregular). (A) Round. (B) Oval. (C) Irregular.

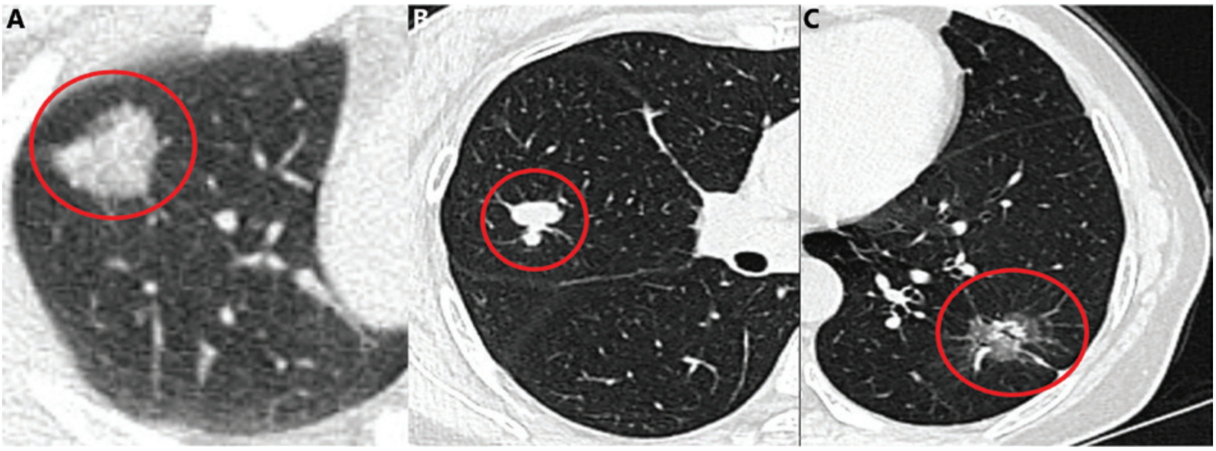

Figure S3 Margins (smooth, lobulated, spiculated). (A) Smooth. (B) Lobulated. (C) Spiculated.

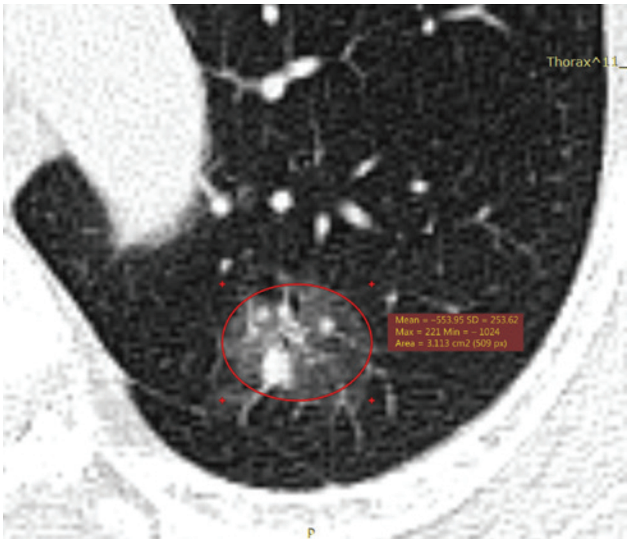

Figure S4 CT density (Hu). CT density (Hu): attenuation values of the largest region of interest in the largest slice of nodule.

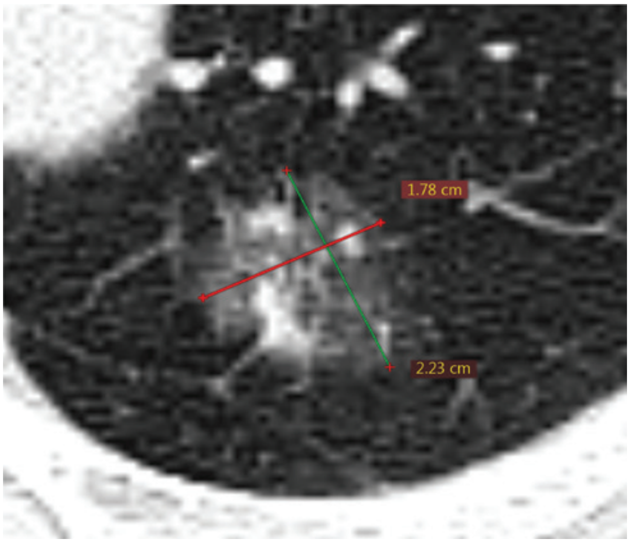

Figure S5 Diameter of the nodule. Diameter: the mean value of largest long diameter and short diameter which measured in lung window setting. Diameter = (22.3 + 17.8)/2 = 20 mm.
